# Challenges to timely access to out-of-hours end-of-life medications: a qualitative study in UK primary care

**DOI:** 10.3399/BJGP.2024.0718

**Published:** 2025-09-09

**Authors:** Ikumi Okamoto, Sophie Pask, Therese Johansson, Rachel L Chambers, Ahmed Mohamed, Philippa G McFarlane, Irene J Higginson, Katherine E Sleeman, Fliss EM Murtagh, Stephen Barclay

**Affiliations:** 1 Primary Care Unit, Department of Public Health and Primary Care, University of Cambridge, Cambridge, UK; 2 Wolfson Palliative Care Research Centre, Hull York Medical School, University of Hull, Hull, UK; 3 Cicely Saunders Institute of Palliative Care, Policy & Rehabilitation, London, UK; 4 King’s College Hospital NHS Foundation Trust, London, UK

**Keywords:** community health services, community pharmacy services, out-of-hours medical care, palliative care, primary health care, qualitative research

## Abstract

**Background:**

Timely, 24/7 access to medications in the community is essential for effective symptom management for people approaching the end of life. Little is known about current systems ensuring skilled and timely patient assessment, prescribing, and administration, and service gaps.

**Aim:**

To investigate out-of-hours access to end-of-life care medications in community settings, identifying gaps, challenges, and potential solutions.

**Design and setting:**

A qualitative study as part of a larger UK research programme concerning out-of-hours community palliative care services.

**Method:**

Structured interviews with key stakeholders responsible for delivering or commissioning palliative care services in the community.

**Results:**

In total, 71 interviews were conducted across 60 areas in the UK. Four key themes were identified: 1) patient assessment and medication prescribing — primary care services are heavily relied on out-of-hours but are often overburdened; 2) dispensing and collecting medications — collecting medications often falls to informal carers, posing challenges, especially in rural areas; in urban settings, safety concerns limit pharmacy opening hours for late-night dispensing of controlled medications; 3) administering medications — this is usually under the responsibility of primary care services, but delays often arise because of overburdened teams; and 4) anticipatory prescribing — the facilitation of timely medication dispensing and collection is limited to identified patients receiving palliative care and is dependent on overburdened community services for administration; concerns over potential drug misuse can occasionally delay anticipatory prescribing.

**Conclusion:**

Systemic challenges and safety concerns hinder timely access to out-of-hours medications for community-based palliative care. Improvements in community pharmacy services, the primary care workforce, and specialist palliative care support are needed.

## How this fits in

Optimal symptom control for people living in the community with advanced illness requires rapid and reliable access to medications around the clock, but obtaining medications in response to changing clinical needs in the community is particularly challenging out-of-hours. While anticipatory prescribing partially addresses these issues, gaps exist in integration, capacity, and equity for timely access. A whole-system approach, avoiding focusing on one part of the process, is needed to improve out-of-hours medication access, along with locally appropriate innovations.

## Introduction

The need for palliative and end-of-life care provision is predicted to increase across the world because of ageing populations and rising rates of chronic illness.^1–3^ To meet this growing demand, palliative care needs to be integrated into primary health care and community settings.^4^ In the UK, deaths in community settings are predicted to double by 2040, highlighting the urgent need for expanded community-based palliative care services.^5^ Similar trends are observed worldwide. ^6–9^

For the successful delivery of community-based palliative care, rapid and reliable access to medication is essential, as it supports symptom control, enhances patient comfort, and reduces avoidable hospital admissions^10^ and associated health service costs. However, achieving optimal symptom management in community settings is a complex process involving multiple professionals^10,11^ and several key stages: 1) patient assessment; 2) medication prescribing; 3) delivery of prescriptions to community pharmacy; 4) pharmacy medication dispensing; 5) delivery of medications to a patient’s home; 6) clinical assessment of medication dose and administration, usually an injection given by a healthcare professional; and 7) reassessment of effect of medicines administration. Efficient and streamlined coordination across these stages is essential for timely symptom control.

People with advanced illnesses commonly experience sudden changes in their symptoms, and much end-of-life care occurs out-of-hours, during evenings, nights, and at weekends, around two-thirds of the week.^12^ This requires around-the-clock care and rapid and reliable access to medications to address emerging symptoms. This is particularly important because, when community-based palliative care fails to meet patients’ needs, the consequences can be significant, adding unnecessary strain to already strained emergency departments and causing distress for both patients and families.^13^

Community-based services are commonly particularly overburdened during out-of-hours periods, making end-of-life care provision especially challenging.^12,14,15^ Furthermore, the availability and accessibility of medications out-of-hours is often problematic.^16,17^ Anticipatory prescribing (Box 1) can be helpful with stages 1–5 above, but is not a panacea.^18^ Not all patients can be identified in advance of need, and, even for identified patients, medication needs can change unpredictably. This study investigated service delivery for out-of-hours access to end-of-life care medication from a whole-system perspective, seeking to identify current gaps and challenges that weaken the process of medication access, explore potential solutions, and highlight practices of potential international applicability.

Box 1. **Key terminology**

‘Anticipatory prescribing’ refers to the prescribing and dispensing of injectable end-of-life medications for patients, in advance of clinical need. These medications are stored at home for trained individuals to administer if the patient experiences symptoms in the final days of life.^39^ ‘Just-in-case prescribing’ and ‘pre-emptive prescribing’ are alternative terms for this practice.

## Method

### Study design

This study, part of a larger study, the ‘Marie Curie Better End of Life Care Programme’, mapped out-of-hours end-of-life care and services available across the four UK nations, using structured qualitative interviews. We present this study following the Standards for Reporting Qualitative Research.^19^

### Participants

To ensure comprehensive regional and national perspectives, we purposively sampled areas across the UK and approached key stakeholders for interview: professionals responsible for palliative and end-of-life care service commissioning or service managers, and senior clinicians and commissioners knowledgeable about out-of-hours services. Participants were selected to represent all four UK nations and diverse areas within each nation. We approached the 42 integrated care systems in England, the seven health boards in Wales, the 14 health boards in Scotland, and the five Health and Social Care Trusts in Northern Ireland (for more detail see Pask *et al* [2024^20^]), followed by snowball sampling.

### Recruitment

An initial invitation was distributed to individuals in each area assisting in the identification of potential participants: a brief overview of the study and a request to contact the research team. The study research associate (second author) responded to interested participants and provided the participant information sheet.

### Data collection

The topic guide was developed from aspects of out-of-hours care previously highlighted as important by patients, informal carers, public representatives, and professionals.^21^ This includes the availability and accessibility of out-of-hours palliative care medications, including the availability of a prescriber out-of-hours, community pharmacies open at night and on weekends and healthcare professionals available to administer medications by injection out-of-hours (Supplementary Box S1).

Interviews were conducted by a non-clinical postgraduate research associate (second author), experienced in qualitative research, who had previous professional acquaintance with two recruited participants. Interviews were held via telephone or video between December 2021 and June 2022, and were audio-recorded with participants’ verbal consent. Four authors transcribed interviews and anonymised transcripts were sent to participants for responder checking.

### Data analysis

This study employed structured interviews, during which participants provided additional information beyond simple closed answers, allowing us to gather rich and detailed data. This qualitative data is presented in the present study. Thematic analysis^22^ was undertaken by the first author with support from two other authors, using an inductive approach. Transcripts were read repeatedly for familiarity and coded using NVivo (version 12) software. The initial coding framework was developed based on the first 10 interviews, with coding refined through extensive discussions with the research team and continuous analysis of the remaining transcripts. No participants provided feedback on the findings.

Findings from the qualitative analysis were discussed in a patient and public contributor workshop in June 2023 involving eight individuals with personal experience of a relative’s end-of-life care at home. Since our interviews were conducted only with professionals, incorporating the viewpoints of these service users enhanced data analysis and interpretation.

## Results

### Participants

**Figure 1.**
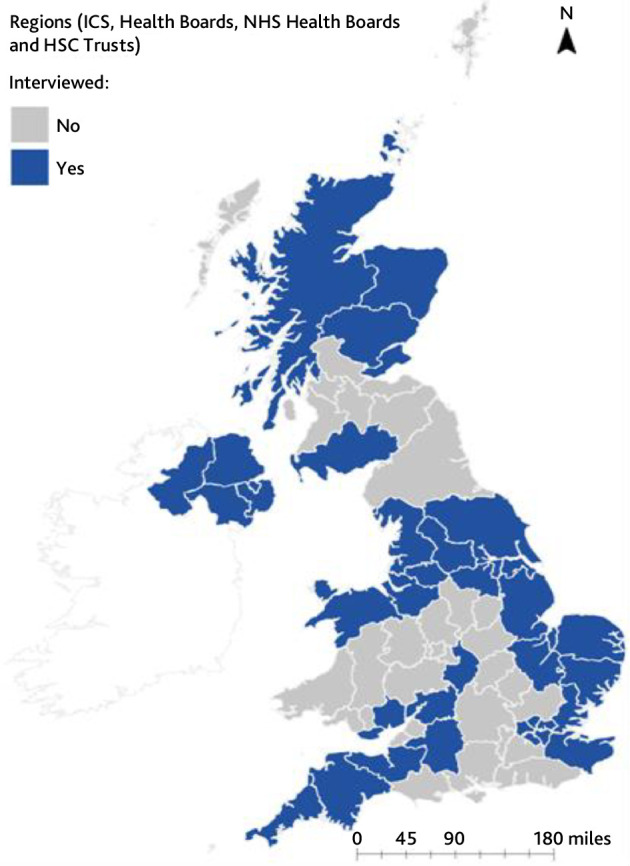
Map of geographical areas covered by interviews (adapted from Pask *et al* ^24^). HSC = Health and Social Care. ICS = integrated care system. N = north.

In total, 71 interviews were conducted with participants from 60 areas across the UK (Figure 1): 27 strategic leads, clinical leads, or managers (oversee healthcare services); 21 commissioners (responsible for planning, funding, and monitoring healthcare services); 36 senior clinicians (including five GPs); and three service development leads. Sixteen participants held multiple roles. All had responsibility for the provision, leading, commissioning, developing, or strategic oversight of out-of-hours services that cared for patients at the end of life. Their average years of experience was 19.3 years (range 3–40 years). Most reported on their local area, though a few reported more widely. Data are presented by geographical area rather than individual participants: for a few areas, there was >1 participant, and their responses were combined into a unified area response. Interviews lasted an average of 39 minutes, with a range of 19 to 72 minutes.

### Thematic analysis

Four main themes were identified (Box 2).

**Box 2.** Summary of themes and sub-themes

Theme	Sub-theme
Patient assessment and medication prescribing	Overburdened community services Early identification of palliative care needs Non-medical prescribers
Dispensing and collecting medications	Designated pharmacies with core end-of-life medications Burden on informal carers Safety concerns with late-night pharmacies Alternative dispensing facilities
Administering medications	Delay in home medication administration Support for medication administration
Anticipatory prescribing	Challenges in timely administration Concerns over drug abuse or theft Balancing safety and timeliness in symptom control

#### Patient assessment and medication prescribing

It was reported that out-of-hours visits are possible in all areas to assess patients and prescribe medication, most commonly from GPs and community (or district) nurses. Community nurses are often the first point of contact: if appropriate medications were not already in place, they would arrange prescriptions with a GP or specialist team. Prescriptions would at times be issued based on a community nurse’s assessment without the prescriber assessing the patient themselves:

*‘*[Healthcare professionals] *would be able to arrange a prescription but they are unlikely to get a visit from a prescriber. Instead, a district nurse would visit them and discuss prescriptions with a GP or the specialist palliative care consultant. If a change in prescription or a new prescription was needed, then that would be accommodated and facilitated.’* (ID48, Wales)

During out-of-hours periods, these services may have long response times because of limited staff covering large geographical areas. Several areas reported that out-of-hours community teams sought to prioritise patients at the end of life.

Obtaining medications out-of-hours is typically easier for patients previously identified as ‘palliative’ in primary care or known to specialist palliative care services; in some areas, these patients are provided with a direct telephone number to community nursing teams or local hospices. Community nurses can sometimes arrange prescriptions via a hospice if the patient is known to the hospice; more commonly, prescribing largely relies on out-of-hours GPs:

*‘The first line is the district nurses; they would have been made aware of the need to assess a patient’s symptoms and then get a prescription. The pathway for the district nurses is to get the prescription from the hospice. If the patient is not known to* [the hospice]*, then it would be more difficult as the hospice will not have access to medical information and may need to involve an out-of-hours GP.’* (ID37, England)

Patients already identified as ‘palliative’ are also more likely to have medications available at home through anticipatory prescribing:

*‘If the patient was formally known to palliative care services; they would already have “just in case” medications prescribed and readily available within their home.’* (ID33, England)

Predicting disease progression, and thus anticipatory prescribing, was reported to be less common for patients without cancer, with potential inequality of medication provision:

*‘They are much better at looking after patients who have cancer because their disease stage is much more predictable. They are not as good at supporting patients living with chronic long-term conditions (such as heart failure, end-stage COPD* [chronic obstructive pulmonary disease]*, neurological disorders, dementia, frailty). This is because they are less confident in classifying these patients as end-of-life.’* (ID30, England)

To reduce medication prescription delays, some areas increased the number of non-medical prescribers, but this must be paired with access to electronic patient records, highlighting the need for coordinated service improvements:

*‘It is challenging to send out a nurse prescriber, especially if they can’t access their previous medical history or recent blood test results to indicate kidney function.’* (ID12, England)

#### Example of good practice and innovation

In one area of England, a hospice service (ID19, England) can prescribe palliative medications following a thorough patient assessment (for example, by a district nurse). This approach proved particularly valuable during the COVID-19 pandemic. While remote prescribing is available between 8.00 am and 6.00 pm, outside these hours, the hospice team provides clinical advice. They also have a high proportion of clinical nurse specialists who are non-medical prescribers, ensuring that a prescriber is almost always available.

#### Dispensing and collecting medications

Prescribed medicines are dispensed by community pharmacies from where they are commonly collected by family members. Many areas had designated pharmacies that stocked core end-of-life medications with lists provided to clinicians to direct patients and carers as needed; however, shortages of core palliative medications were at times reported:

*‘There is a list of pharmacies that stock palliative medicines and a rota of pharmacies open 24-hours, which means they could dispense medicines overnight, on the weekend, or bank holidays. The CCG* [clinical commissioning group] *commissions specific pharmacies to hold end-of-life medications. However, there have been instances where these commissioned pharmacies do not have end-of-life medications in stock.’* (ID4, England)

Some participants also indicated the lack of updated information on the availability of out-of-hours community pharmacies for patients, carers, and healthcare professionals:

*‘The specialist team should have access to information of pharmacies that hold controlled drugs. But this is difficult to maintain, perhaps due to communication.’* (ID17, England)

Family members may have to travel long distances to collect medications, especially in rural areas, which can be both time consuming and burdensome:

*‘Pharmacy is highlighted as an issue in the area because of the rurality.* […] *Families have been known to drive around the county to try and source medication which can take many hours.’* (ID17, England)

In some areas there were concerns regarding the safety of late-night pharmacies dispensing controlled medications:

*‘They previously tried to set up an out-of-hours pharmacy rota, but this was very difficult to put in place. One of the resounding issues was safety and security with a pharmacy being open in the middle of the night.’* (ID35, England)

To improve the process of dispensing and collecting medications out-of-hours, several areas had commissioned alternative dispensing facilities, including hospital and hospice pharmacies:

*‘There is a pharmacy which is open on weekends/bank holidays: if there isn’t one available then there is an agreement that the hospital will issue the prescription.’* (ID42, England)

Furthermore, in some areas, out-of-hours GPs or ambulance paramedics carry supplies of core medications:

*‘GPs carry routine medications. Therefore, they do not need access to an out-of-hours pharmacy. The GPs do not carry less common medications but can access these through the hospital or use alternatives until the morning.’* (ID54, Scotland)

In some areas, healthcare professionals or driver services are available to assist patients and carers with medication collection:

*‘There is a protocol in place …* [if] *families cannot collect the medication, and it’s not feasible for a district nurse to collect the medication at night, then there is a driver service.’* (ID37, England)

Participants also suggested increasing the number of community pharmacies open 24/7 and ensuring the availability of core end-of-life medications in pharmacies to improve the medication dispensing and collecting process.

##### Example of good practice and innovation

In an urban area of England (ID35, England), prescriptions held by the patients and family could not be dispensed out-of-hours. A GP cooperative established a protocol for obtaining palliative medications from the local hospital’s out-of-hours pharmacy and delivering them to patients' homes. A nurse and GP are often present in a fleet car and administer the medications. This approach not only reduces the burden on family members or friends who would otherwise need to collect medication, but also ensures timely administration and improves the safety and security of both healthcare staff and family members/friends.

### Administering medications

A visit from a community nurse is usually needed for medication administration by injection. These services are available 24/7 in most (but not all) areas, but are often under significant demand, leading to long waiting times:

*‘There are people who can administer palliative medicines, but the geography may be challenging, i.e., what is the out-of-hours nurse or doctor doing at that time and what distance they have to travel, and there may be varying demand for the service.’* (ID53, Scotland)

Timely administration of medications depends not only on obtaining them promptly but also on the availability of healthcare professionals who can visit patients and administrate them quickly. In some areas, ambulance or rapid response teams are available to visit and administer medications:

*‘The administration of medications would be done by the rapid response team, which is the joint hospice and NHS service. They can respond within 30 minutes, depending on geography.’* (ID9, England)

In a few areas, informal carer administration had been introduced to address delays in community nurse visits, with a strict protocol determining suitable family members, sometimes restricted to those with a healthcare background:

*‘*[There is] *also a system, where they teach relatives to administer medications, so they can administer out-of-hours. This is usually for patients who are predictably unpredictable with their pain and have variable analgesic needs. It is not suitable for everybody and there is a strict protocol to determine whether people are suitable to be trained for this.’* (ID47, Wales)

#### Example of good practice and innovation

A Specialist Palliative Care Paramedic Service operates independently from the standard ambulance service, providing home visits to patients requiring palliative care (ID49, Wales). These paramedics can administer medication if it is already available in the home, but plans are being developed to allow them to carry anticipatory medicines. This initiative enhances responsiveness by offering an additional layer of support alongside existing community services, ensuring that patients receive timely medication administration. Furthermore, as these paramedics are specifically trained in palliative care, they can provide more specialised support for patients at the end of life.

### Anticipatory prescribing

There was broad support for anticipatory prescribing as a way to address the difficulties of accessing medications out-of-hours:

*‘Pharmacies are not necessarily open throughout the night. This is a challenge and is why anticipatory medications are available.’* (ID56, Northern Ireland)

While anticipatory prescribing addresses the prescribing, dispensing, and collecting of medicines, timely administration remained a challenge:

*‘Anticipatory medications are often in place but there isn’t anyone to give them. It would be better to have a 24/7 Community Nursing Service* [which is not in place]*. Prescriptions and plans are in place but often there is no one to administer them.’* (ID17, England)

In some areas, there were concerns over drug abuse or theft that was often addressed by medications being kept in locked boxes or being issued later than normal as ways to balance medication safety and symptom control:

*‘Pre-emptives are issued quite late and there are people who should have had medications in place.* […] *Instances where people have misused pre-emptives in the past have made* [healthcare professionals] *more wary about having them in the house too soon.’* (ID32, England)

Mixed views were reported on anticipatory prescribing of syringe drivers, at times with conflicting views within the same area:

*‘The community provider has a policy to prescribe syringe drivers in anticipation, which the specialist palliative teams don’t agree with and don’t feel is safe, as the driver should be considered and assessed in the moment. The GPs do prescribe syringe drivers in advance, but the specialist teams wouldn’t do this out of the hospice or the hospital.’* (ID21 and ID36, England)

### Reflections from the patient and public contributor workshop

Our patient, family carer, and public contributors highlighted that end-of-life medication management needed to be integrated throughout all stages: minor deficiencies can cause significant delays and avoidable suffering.

Family members often need to collect medication from community pharmacies, which is particularly problematic in rural areas, for those dependent on public transportation, women travelling alone at night, and those with other caregiving responsibilities. It was distressing to leave loved ones in pain at home while collecting medications, to encounter long waits for medication administration, and to experience difficulties in contacting services for help. Family carers expressed feelings of helplessness and guilt when there were system failures or insufficient services.

## Discussion

### Summary

This study identified numerous challenges in the complex process of out-of-hours medication access for those with palliative and end-of-life care needs in the community, from patient assessment, to prescribing, dispensing, collection, and administration of medications. The entire process must work cohesively and in an integrated manner: the chain is as strong as its weakest link. Key issues include overburdened community nursing and primary care services, a reliance on prognosis-based palliative care provision, the demands on informal carers, limited pharmacy opening hours, delays in medication administration, and safety concerns. Addressing these issues is an urgent priority, as it will help in establishing seamless, timely, and safe processes for out-of-hours end-of-life medications.

### Strengths and limitations

By exploring the perspectives of professionals involved in delivering or commissioning community-based palliative care, this study highlights not only how palliative medication is accessed during out-of-hours periods, but also highlighted the significance of service coordination at a systemic level. Interviews with professionals across different regions in the UK revealed variations in service delivery, and localised strategies to meet the specific needs of each area.

Focusing on systemic aspects of service delivery, interviews were conducted only with professionals who have clinical and commissioning responsibilities in palliative and end-of-life care. While we did not obtain the perspectives of patients and family members, our patient and public contributor workshop brought their lived experiences to our data analysis and interpretation.

The detailed qualitative data provides nuanced insights into complex issues; however, the structured interview format may have limited opportunities for participants to respond in great depth.^23^

Despite our efforts to recruit participants from across all UK regions, some geographical areas did not participate, potentially limiting the full capture of variability in out-of-hours palliative medication access and the generalisability of findings.

This study focuses on qualitative insights into out-of-hours medication management. For the quantitative aspect of this study, see Pask *et al* (2022).^24^

### Comparison with existing literature

Our findings reinforce and expand on previous research on the difficulties encountered in accessing end-of-life medications out-of-hours. For instance, Latter *et al* (2022) reported similar issues, such as medications not being ready for pickup or being out of stock (requiring repeat pharmacy trips), miscommunications between pharmacies and prescribers, limited opening hours, long pharmacy wait times, difficulties leaving patients unattended to collect medications, and travel burdens.^11^ While our study confirms these barriers, it further contributes by demonstrating regional variations in medication access challenges and identifying local solutions. Moreover, our whole-system analysis highlights the importance of a cohesive and integrated approach across the entire process. Our patient and public contributor workshop revealed how informal carers’ experiences of and satisfaction with end-of-life care provision impact their bereavement experience, highlighting the importance of recognising the long-term effects on carers and the pressing need to alleviate their burden.

Community pharmacies have a central role,^25^ but face limitations regarding timely access to medications due to shortages of palliative medications. Contributing factors include difficulties in forecasting demand, concerns about medication expiry dates, complexities in navigating the interface with wholesalers and distributors, and pharmacy storage space limitations.^26^ Our findings indicate that some areas have implemented strategies to reduce reliance on out-of-hours pharmacies, including alternative dispensing facilities and equipping out-of-hours GPs or ambulance paramedics with core medications. To help informal carers, some areas provide healthcare professionals or driver services for medication collection and delivery.

To speed up prescribing, some surveyed areas had expanded non-medical prescribing; however, implementation was sometimes hindered by limited access to electronic medical records.^11,27,28^ Our findings also indicate that community nurses, who are often the first point of contact, would arrange prescriptions with a GP or specialist team.

Patients identified as ‘palliative’ or known to specialist palliative care had easier medication access out-of-hours, including direct telephone numbers to healthcare professionals and greater probability of anticipatory medications being in place. Timely identification of palliative care needs has previously been described as a *‘first-class ticket*’ to enhanced community end-of-life care.^29^ However, the identification of people approaching the end of life, endorsed in UK national guidance,^30^ is highly variable.^29,31,32^

While timely palliative care need identification is important for community-based patients to access medication out-of-hours, difficulty in predicting disease progression in patients without cancer was reported, which often leads to less frequent anticipatory prescribing and poorer access to end-of-life care medications.^33^ A greater focus on present and future symptom management needs rather than prognosis enables better timing of anticipatory medicines.^34^

While 24/7 community nurse home visits are, in theory, available in almost all UK areas, these services are often under significant pressure out-of-hours, leading to frequent medication administration delays. While prompt response from ambulance or rapid response teams is beneficial for symptom control and provides an additional sense of safety and security to patients and informal carers,^35^ our study revealed that, in most areas, patients still have to rely on overburdened community nurses for medication administration.

When community-based palliative care fails to meet patient needs, the impact extends beyond clinical delays, leading to emergency hospital admissions,^36–38^ increased healthcare costs,^37^ and distress for patients and families.^13^ While our study did not directly evaluate these impacts, these broader consequences highlight the need for improved community out-of-hours palliative care service.

Anticipatory prescribing was used in all participating areas and is widely available in the UK.^39,40^ While this helps to address prescribing, dispensing, and collecting of medicines,^16,39^ our study highlighted long wait times for (or even unavailability of) community nurses or other healthcare professionals to administer medicines. A small number of areas reported that informal carer medication administration was in place in order to reduce these delays (for a practical example of such an approach, see the CARiAD package^41^).

Varied views concerning anticipatory prescribing of syringe drivers have been previously reported, with concerns over the inability to anticipate dosage or drug changes between initial prescribing and administration.^42^ The need to balance symptom control and patient safety is a continuing tension. Previous research has also reported concerns over potential drug misuse in relation to anticipatory prescribing, leading to later prescribing.^43^

### Implications for research and practice

There is a need for systemic change and collaborative efforts among healthcare providers, service managers, and commissioners to enable seamless medication access for people with advanced illness during out-of-hours.

Out-of-hours access to end-of-life care medications relies heavily on primary care teams, particularly community nurses, who often experience delays in patient assessment because of heavy workloads. Increased numbers of non-medical prescribers may help address prescribing delays, provided patient records are effectively shared. Involving other healthcare professionals, such as rapid response teams, and ensuring specialist palliative support is available to community teams out-of-hours may facilitate more timely patient assessment and prescribing, although more evidence is needed.

The challenges in dispensing and collecting medications highlight a need for around-the-clock community pharmacy services in all areas. Additional dispensing facilities, such as hospital or hospice pharmacies and approved courier services, could improve access and reduce the burden on informal carers, particularly in rural areas. Service commissioners should consider piloting these approaches to assess their feasibility and cost-effectiveness.

Delays in medication administration are often linked to limited out-of-hours community nursing services. Expanding staffing and resources of out-of-hours primary care could help ensure that patients receive medication promptly. Further research should evaluate the effectiveness of alternative models, including the involvement of rapid response teams, ambulance crews, or protocols for training selected informal carers to ensure safe and timely administration.

While anticipatory prescribing may reduce medication access delays, its effectiveness relies on timely administration by healthcare professionals. Our findings suggest that anticipatory prescribing protocol should be refined to ensure effective administration. In some settings, additional safeguards protocol may be necessary to address risks such as drug misuse. Anticipatory syringe driver prescribing remains controversial and requires further study. As anticipatory medications are primarily available to formally identified patients receiving palliative care, guidelines and decision tools, such as EARLY,^44^ are needed to facilitate early identification of palliative care needs regardless of diagnosis.

Future studies should also address the priorities and experiences of both patients and informal carers to allow service providers to tailor the services to users' values and preferences.^45^
